# LncRNA81246 regulates resistance against tea leaf spot by interrupting the miR164d‐mediated degradation of NAC1

**DOI:** 10.1111/tpj.17173

**Published:** 2024-11-26

**Authors:** Di Guo, Dongxue Li, Fenghua Liu, Yue Ma, Jing‐Jiang Zhou, Sujitraj Sheth, Baoan Song, Zhuo Chen

**Affiliations:** ^1^ State Key Laboratory of Green Pesticide, Key Laboratory of Green Pesticide and Agricultural Bioengineering, Ministry of Education Guizhou University Guiyang Guizhou 550025 China; ^2^ College of Tea Science Guizhou University Guiyang Guizhou 550025 China; ^3^ College of Agriculture Guizhou University Guiyang Guizhou 550025 China; ^4^ Medical Research Council Mitochondrial Biology Unit University of Cambridge Cambridge CB2 0XY UK

**Keywords:** noncoding RNA, ceRNA, *CsNAC1*, tea leaf spot, disease resistance, miR164, long noncoding RNA, microRNA

## Abstract

Non‐coding RNAs play crucial roles in plant responses to viral stresses. However, their molecular mechanisms in tea leaf spot responses remain unclear. In this study, using *Camellia sinensis*, we identified lncRNA81246 as a long non‐coding RNA that localizes to both the nucleus and cytoplasm. It functions as a competitive endogenous RNA, thereby disrupting *CsNAC1* (encoding NAC domain‐containing protein 1) degradation mediated by miR164d. Silencing lncRNA81246 increased the resistance of tea plants to presistanceathogens, whereas transient lncRNA81246‐overexpression plants showed decreased resistance to pathogens. Co‐expression assays in *Nicotiana benthamiana* revealed that lncRNA81246 affects the miR164d–*CsNAC1* regulatory module. Transient miR164d‐overexpression and silencing assays demonstrated its positive regulation of tea plant resistance. Specifically, silencing its target, *CsNAC1*, enhanced disease resistance, whereas transient overexpression reduced plant resistance. Yeast one‐hybrid, dual‐luciferase, and RT‐qPCR assay results suggested that CsNAC1 alters the expression of *CsEXLB1*, whereas AsODN and tobacco transient overexpression assays showed that *CsEXLB1* negatively regulated tea plant resistance. Thus, our research demonstrated that lncRNA81246 acts as a mediator to interfere with the miR164d–*CsNAC1* regulatory module involved in the disease resistance of tea plants.

## Significance Statement

It was first discovered that the competing endogenous RNA module involving LncRNA81246, miR164d, CsNAC1, and CsEXLB1 is associated with the disease resistance of tea leaf spot caused by the hyphae of Lasiodiplodia theobromae.

## INTRODUCTION

Non‐coding RNAs (ncRNAs), comprising long non‐coding RNAs (lncRNAs), microRNAs (miRNAs), and circular RNAs, are special types of RNAs that lack the capability to encode proteins (David, 2004; Palos et al., 2023; Yu et al., 2019). With advancements in high‐throughput sequencing technology, these ncRNAs were found to play indispensable roles in diverse biological processes, such as development, cell differentiation, and both abiotic and biotic stress responses (Feng et al., 2014; Samarfard et al., 2022; Wang et al., 2017; Wang, Luo, et al., 2018).

LncRNAs, which exceed 200 nucleotides in length, form a diverse group of transcripts that play significant roles in plants (Palos et al., 2023). For instance, salicylic acid biogenesis controller 1 modulates salicylic acid biosynthesis, resulting in a balance between plant defense and growth (Liu et al., 2022). In tomato (*Solanum lycopersicum*), lncRNA16397, an antisense transcript of *glutaredoxin 22* induces *glutaredoxin* expression to negatively regulate the accumulation of reactive oxygen species, resulting in enhanced resistance to *Phytophthora infestans* (Cui et al., 2017). ELF18‐INDUCED LONG NONCODING RNA 1, as an lncRNA induced by bacterial flagellin or translation elongation factor Tu, increases the expression of *pathogenesis‐related protein 1* and enhances the resistance of *Arabidopsis thaliana* (Seo et al., 2019).

As a crucial type of noncoding RNA, miRNAs encoded by *MIR* genes also play indispensable roles in plant defense mechanisms (He et al., 2022). In rice (*Oryza sativa*), the miR164a–*OsNAC60* regulatory module manipulates defense responses to blast fungus *Magnaporthe oryzae* infection (Wang, Xia, et al., 2018). Osa‐miR167d negatively regulates rice immunity to facilitate *M. oryzae* infection by downregulating the expression of *OsARF12* (Zhao et al., 2020). Overexpressing Osa‐miR1873 enhances the susceptibility to *M. oryzae* and compromises the induction of defense responses by regulating LOC_Os05g01790 (Zhou et al., 2020). Blocking Osa‐miR1871 enhances the expression of *microfibrillar‐associated protein*, which is located near the cell wall and positively regulates pattern‐triggered immunity responses (Li, Li, et al., 2022; Li, Ye, et al., 2022).

LncRNAs contain miRNA‐recognition elements, allowing them to act as miRNA sponges that remove the inhibitory effects of miRNAs on their target genes (Ebert et al., 2007; Leonardo et al., 2011; Marcella et al., 2011). This regulatory mechanism, referred to as competing endogenous RNAs (ceRNA) modules, has been increasingly recognized for its involvement in the physiological and biochemical regulation within plants (Tay et al., 2014). In tomato, Sly‐lncRNA15492 and lncRNA23468 competitively bind with Sly‐miR482a and miR482, altering the expression of *nucleotide‐binding site leucine‐rich repeat 1* against *P. infestans* (Jiang et al., 2019, 2020). Overexpressing lncRNA39026 increases the level of *argonaute*, which is targeted by miRNA168, and enhances the resistance to *P. infestans* (Hou et al., 2019). The tomato resistance to *P. infestans* is negatively regulated by the lncRNA39896–miR166b–*HDZs* module (Hong et al., 2022). In upland cotton, lncRNA354 acts as the endogenous target mimic of miR160b to regulate *ARF17/18* in response to salt stress (Zhang et al., 2021). In maize (*Zea mays*), the module regulating miRNA399–*ZmPHT1* is influenced by the lncRNA PILNCR2 in response to low‐phosphorus stress (Wang et al., 2023).

The buds and leaves of tea (*C. sinensis* (L.) O. Kuntze) are often processed into beverages that are highly economically profitable. Because of an increase in disease incidence and severity, as well as a shortage of control measures, tea leaf spot caused by pathogens can result in huge losses of tea leaves, resulting in decreased production and profit (Deng et al., 2023; Li et al., 2019). The involvement of mRNAs and ncRNA interactions are indispensable in conferring resistance traits to tea plants (Jeyaraj et al., 2019; Wang et al., 2021). We observed previously that NAC (NAM, ATAF, and CUC) transcription factors play crucial roles in response to *Lasiodiplodia theobromae* infection. NAC transcription factors participate in stress responses, nutrient metabolism, and cell differentiation (Chen et al., 2011; Han et al., 2023; Yuan et al., 2019). NAC2c promotes resistance to *Ralstonia solanacearum* infection and interacts with HSP70 to regulate immunity in pepper (Cai et al., 2021). In *Arabidopsis*, the genes *AtATAF1* and *AtATAF2* negatively regulate plant resistance to both necrotrophic fungi and bacteria, with AtATAF2 specifically regulating resistance against *Fusarium oxysporum* (Delessert et al., 2005; Wang et al., 2009). However, the molecular mechanisms underlying NAC functions in tea plant are still not well understood.

The crucial roles of ceRNA modules in regulating plant resistance have been demonstrated, but research on the responses of tea plant ceRNAs to pathogens remains limited. Numerous lncRNAs and miRNAs mediate defenses against tea leaf spot (Jiang et al., 2021; Li et al., 2021). Candidate miRNA–mRNA and lncRNA–miRNA–mRNA regulatory modules preventing tea leaf spot have been predicted and analyzed (Guo et al., 2022). In this study, we discovered a novel lncRNA, lncRNA81246, which interacts with miR164d, thereby interfering with the cleavage of *CsNAC1* transcripts by miR164d. Moreover, CsNAC1 negatively affected tea plant resistance to pathogens, whereas miR164d enhanced plant resistance to pathogens by degrading *CsNAC1*. Furthermore, silencing lncRNA81246 enhanced disease resistance, whereas transient overexpression boosted pathogen infection. These findings contribute to our comprehension of the intricate molecular mechanisms involved in plant immunity and highlight the potential of ncRNAs as promising targets for enhancing plant disease resistance.

## RESULTS

### LncRNA81246 functions as an ncRNA

**Figure 1:**
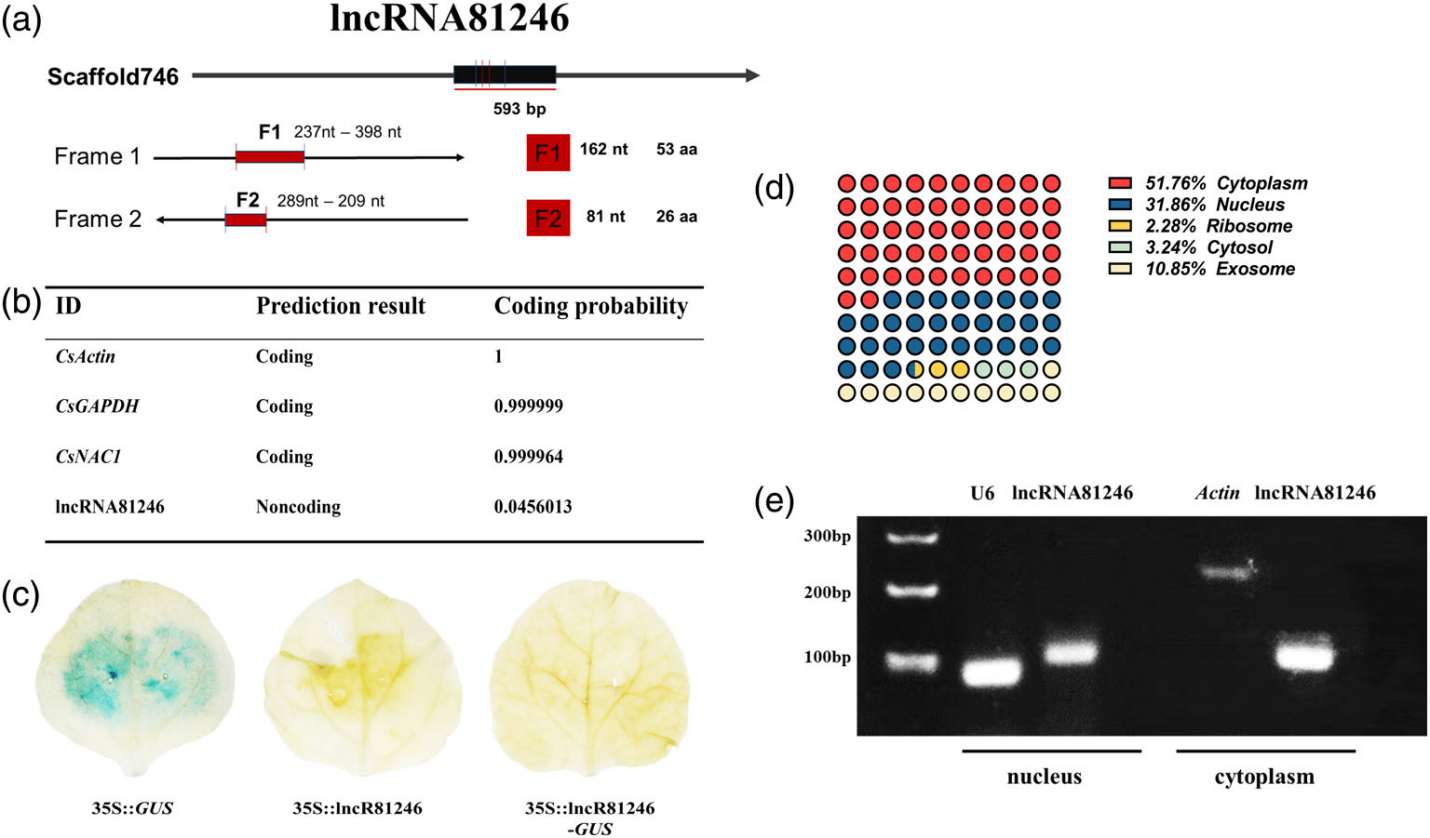
The coding potential and subcellular localization analysis of lncRNA81246. (a) Analysis of the lncRNA81246 ORF. Two frames (red boxes) were identified. The two longest ORFs were predicted to encode polypeptides of 53 and 26 aa. (b) Analysis of the coding potential of lncRNA81246. Coding potential scores were generated using the CPC2 program. *Actin*, *GAPDH*, and *CsNAC1* were used as positive controls. (c) pBI121‐lncRNA81246‐GUS vectors were constructed, and transformed into tobacco leaves. *β*‐Glucuronidase (GUS) staining was used to show the potential coding capacity. (d) The subcellular localization of lncRNA81246 as predicted by lncLocator2.0 online tools. (e) Detection of the subcellular locations of lncRNA81246 by RT‐PCR of nuclear and cytoplasmic extracts. U6 and *β*‐*actin* were used as nuclear and cytosolic RNA markers, respectively.

In a previous study, a significant decrease in the expression of lncRNA81246 occurs after infection by *L. theobromae*, whereas the expression of miR164d increases significantly (Guo et al., 2022). Furthermore, *CsNAC1*, which is the target gene of miR164d, experienced a significant reduction in its expression (Table S1). To determine whether lncRNA81246 is a non‐coding RNA, the open reading frames of lncRNA81246 were verified using ORF Finder online tools (https://www.ncbi.nlm.nih.gov/orffinder/). The results showed that the two longest encoded peptides were 53 and 26 amino acids (Figure 1a). In addition, CPC2 online tools (https://cpc2.gaolab.org/) were used to calculate the lncRNA81246 coding ability and indicated that lncRNA81246 is an ncRNA (Figure 1b). *β*‐Glucuronidase (GUS) histochemical staining was further employed to verify the coding ability, the constructed expression vector 35S::lncRNA81246‐GUS revealed no GUS activity (Figure 1c). Therefore, we concluded that lncRNA81246 is an ncRNA. Subcellular localization is a critical factor for lncRNA functions; therefore, we used lncLocator2.0 (https://www.csbio.sjtu.edu.cn/bioinf/lncLocator2/) to predict the subcellular localization of lncRNA81246 (Lin et al., 2021). The prediction results indicated that lncRNA81246 is distributed in both the cytoplasm and nucleus (Figure 1d). Subsequently, nuclear and cytoplasmic extract assays were conducted to verify the subcellular localization of lncRNA81246, which further confirmed its presence in both the cytoplasm and nucleus (Figure 1e). The subcellular localization of lncRNA81246 indicated the possibility of ceRNA regulatory mechanism.

### LncRNA81246 regulates tea plant disease resistance

**Figure 2:**
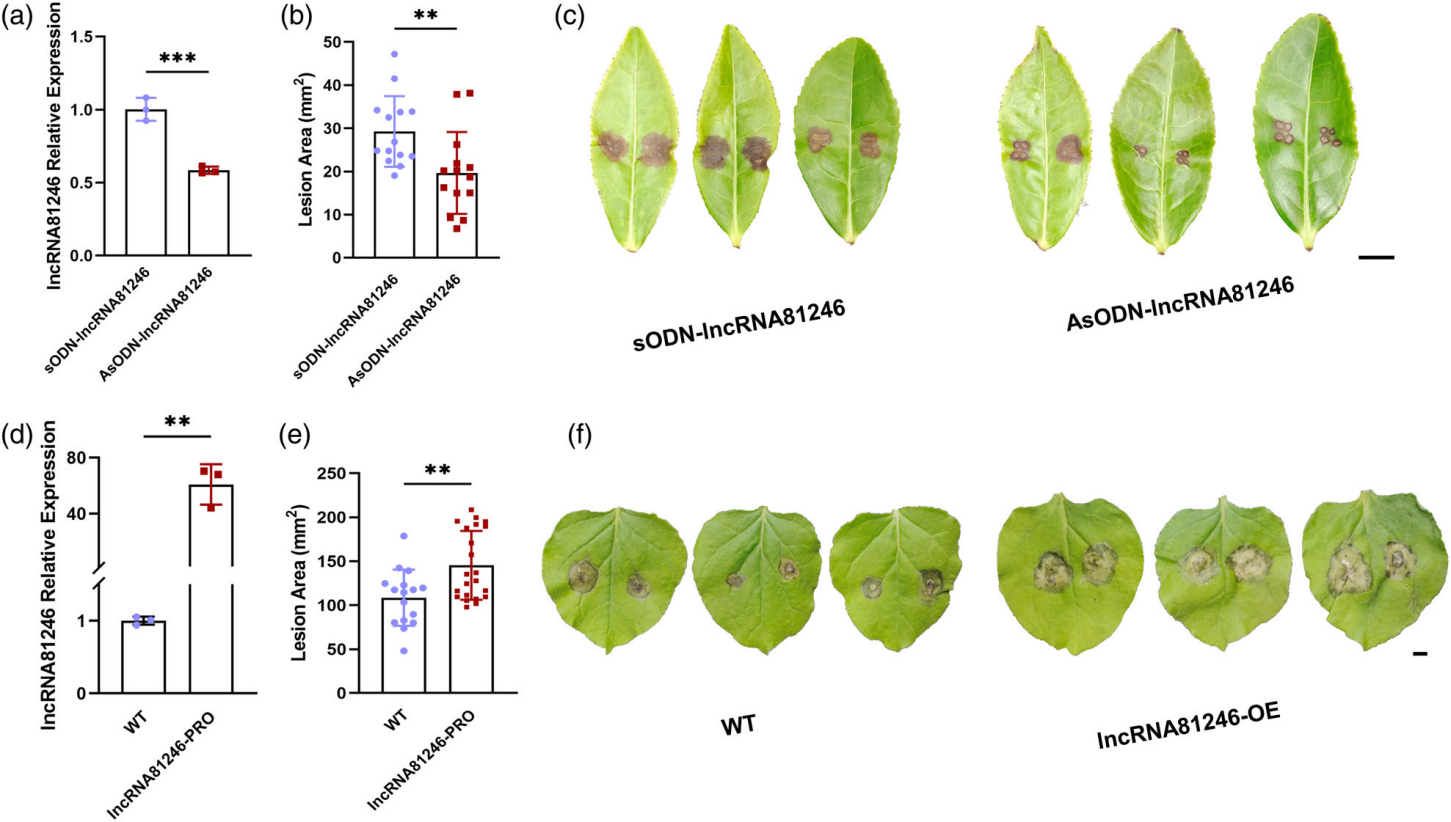
AsODN of lncRNA81246 in tea leaves and transient lncRNA81246 expression in tobacco leaves. (a) Relative expression levels of lncRNA81246 after independent treatment with sODN‐lncRNA81246 and AsODN‐lncRNA81246. (b) The lesion areas of tea leaves incubated independently with sODN‐lncRNA81246 and AsODN‐lncRNA81246 at 12 h after pathogen infection treatments. (c) The phenotypes of tea leaves incubated independently with sODN‐lncRNA81246 and AsODN‐lncRNA81246 at 12 h after pathogen infection. Scale bar = 1 cm. (d) The relative expression levels of lncRNA81246 in wild‐type and in lncRNA81246‐overexpressed tobacco leaves. (e) The lesion areas of wild‐type and lncRNA81246‐overexpressed tobacco leaves at 12 h after pathogen infection. (f) The phenotypes of wild‐type and lncRNA81246‐overexpressed tobacco leaves at 12 h after pathogen infection. Scale bar = 1 cm. RT‐qPCR quantifications were normalized to the expression of *GAPDH* and U6 of tea plant, and U6 and *UBC* of tobacco. Error bars represent standard errors (*n* ≥ 3). Asterisks represent statistically significant differences (*P < 0.05, **P < 0.01, ***P < 0.001) as determined by Student's *t*‐tests.

To investigate the role of lncRNA81246 in regulating tea plant defenses against disease, lncRNA81246 was suppressed in tea leaves using an antisense oligonucleotide (AsODN), with the sense oligonucleotide (sODN) serving as a control. The RT‐qPCR results demonstrated the successful silencing of lncRNA81246 (Figure 2a). An analysis of the lesions on tea leaves infected by *L. theobromae* hypha revealed that the lesion area was significantly reduced in tea leaves having lncRNA81246 silenced by AsODN compared with those treated with sODN. This suggested that lncRNA81246 negatively affected the disease resistance of tea leaves (Figure 2b,c). To further uncover the disease resistance mechanism of lncRNA81246, a 35S::lncRNA81246‐overexpression vector was constructed. Previous assays revealed the high conservation of miRNAs across different species; consequently, we hypothesized that lncRNA82146 regulates disease resistance through a miRNA–mRNA module. We transiently overexpressed lncRNA81246 in tobacco leaves, then confirmed successful overexpression through a RT‐qPCR analysis (Figure 2d). An evaluation of the leaf pathogenicity using the inoculation of *Botrytis cinerea* hypha revealed a significant increase in lesion area compared with the wild type (WT) (Figure 2e,f). Silencing and transient overexpression experiments with lncRNA81246 suggested its involvement in tea plant and tobacco disease resistance.

### LncRNA81246 interacts with miR164d to regulate *CsNAC1* expression

**Figure 3:**
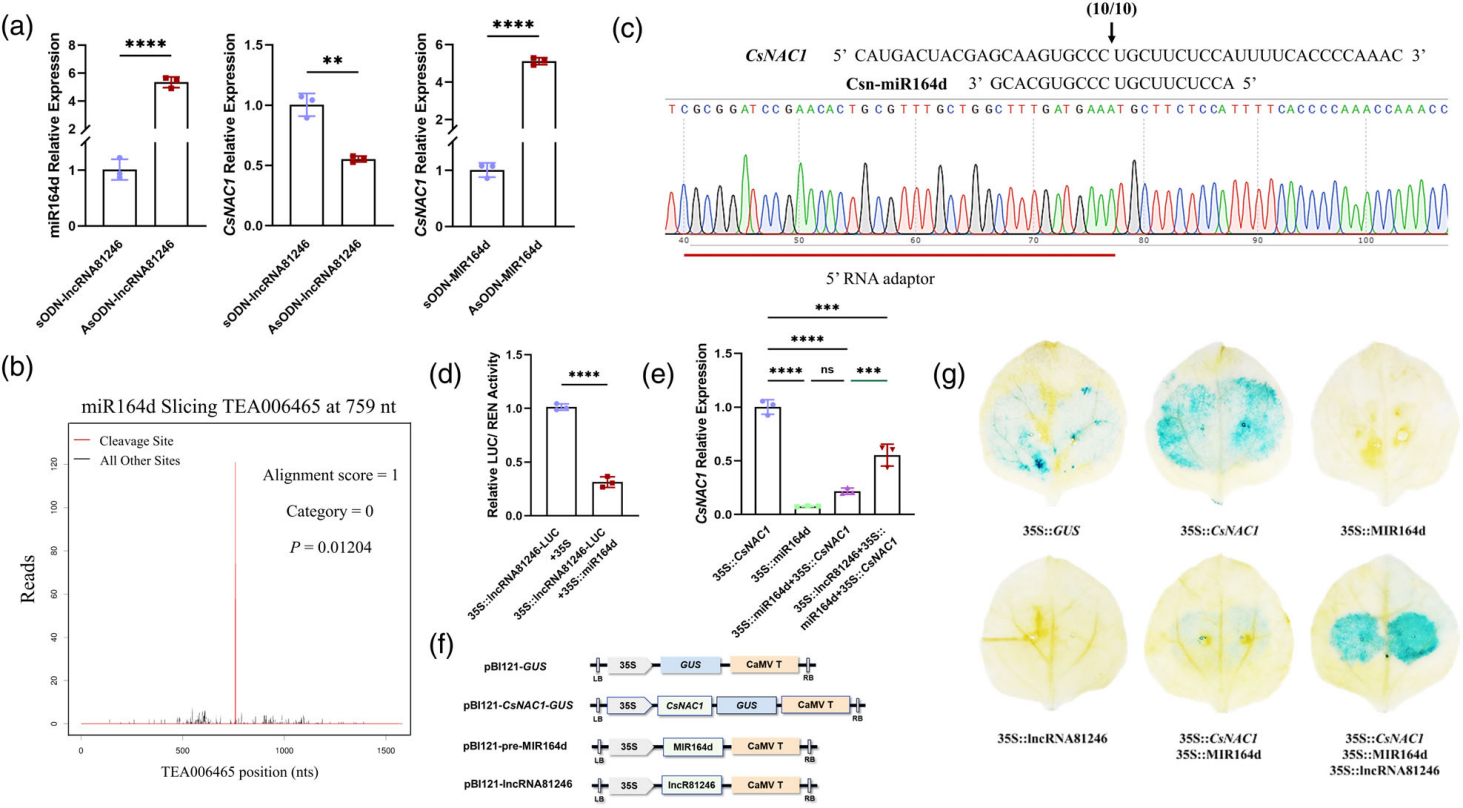
Regulatory relationships among lncRNA81246, miR164d, and *CsNAC1*. (a) Relative expression levels of miR164d and *CsNAC1* after treatment with AsODN‐lncRNA81246 and AsODN‐miR164d. (b) The degradome sequencing showed that miR164d targeted and cleaved *CsNAC1* at the 759 nt. Category = 0 means raw data fragment at this position with an abundance equal to the maximum abundance of this transcribed RNA and with only 1 maximum value. (c) 5′ RLM‐RACE validated the relationship between *CsNAC1* and miR164d. The arrows indicate the cleavage sites. (d) Effects of miR164d on the lncRNA81246 were determined using LUC/REN activity in *N. benthamiana* leaves. The LUC/REN ratio of the empty vector (SK) was used for normalization. Mean values with SDs are shown for three biological replicates. (e) Relative expression level of *CsNAC1* in tobacco after co‐expression treatments with 35S::*CsNAC1*, 35S::pre‐miR164d, and 35S::lncRNA81246 in tobacco using RT‐qPCR. (f) Schematics of pBI121::*GUS*, pBI121::*CsNAC1*‐*GUS*, pBI121::pre‐miR164d, and pBI121::lncRNA81246 vectors. (g) *β*‐Glucuronidase (GUS) staining was used to show the relationships among lncRNA81246, miR164d, and *CsNAC1*. 35S::*GUS* was used as a positive control; 35S:: miR164d and 35S::lncRNA81246 were used as negative controls. *UBC*, U6, and *GAPDH* were used to normalize the *CsNAC1* expression level in tobacco, and the miR164d expression level and *CsNAC1* expression level in tea leaves, respectively. Error bars represent standard errors (*n* = 3). Asterisks represent statistically significant differences (ns >0.05, **P < 0.01, ***P < 0.001, ****P < 0.0001) as determined by Student's *t*‐tests.

In previous study, we found a potential ceRNA regulatory mechanism among lncRNA81246, miR164d, and *CsNAC1*. We analyzed the expression levels of downstream related genes in silenced samples treated with AsODN‐lncRNA81246. Upon silencing lncRNA81246, there was a significant increase in miR164d expression, whereas the expression of *CsNAC1* decreased by half. Additionally, there was a significant up‐regulation of *CsNAC1* expression in tea leaves treated with AsODN‐miR164d (Figure 3a). Ssearch36 software (version 36.3.6) was used to predict the interaction between lncRNA81246 and miR164d. In addition, TargetFinder (https://github.com/carringtonlab/TargetFinder) was then used to predict the cleavage site on *CsNAC1* mRNA of miR164d. The results inferred that lncRNA81246 was able to interact with miR164d at the range 332 to 355 bp of lncRNA81246, thereby interfering with the cleavage of *CsNAC1* by miR164d (Table S1, Figure S6).

To verify the cleavage of *CsNAC1* mRNA by miR164d, degradome sequencing was further used to confirm the interaction between miR164d and *CsNAC1* mRNA and revealed the miR164d cleaved the mRNA of *CsNAC1* at the 759th nucleotide (Figure 3b). Subsequently, a modified 5′ RLM‐RACE experiment was employed to verify the cleavage site of *CsNAC1* by miR164d. The result showed that *CsNAC1* was cleaved by miR164d between the 10th and 11th nucleotides at the 5′ end of miR164d, consistent with the findings of the degradome sequencing assay (Figure 3c). For validation of lncRNA81249 and miR164d interaction, a dual‐luciferase assay was performed using 35S::lncRNA81246‐LUC and 35S::pre‐miR164d. When 35S::lncRNA81246‐LUC and 35S::pre‐miR164d were co‐injected into *Nicotiana benthamiana* leaves, miR164d can bind the sequence of lncRNA81246 upstream of the *LUC* gene, thereby interfering with *LUC* transcription. So, miR164d significantly decreased the expression of *LUC*, with a lower LUC/REN ratio. This supports that lncRNA81246 can bind miR164d (Figure 3d).

Subsequently, three overexpression vectors, 35S::*CsNAC1*, 35S::miR164d, and 35S::lncRNA81246, were individually or co‐transformed into *N. benthamiana*. The co‐transformation of 35S::*CsNAC1* and 35S::miR164d into tobacco leaves significantly reduced *CsNAC1* expression levels compared with the transformation of 35S::*CsNAC1* only. When the three overexpression vectors 35S::*CsNAC1*, 35S::miR164d, and 35S::lncRNA81246 were co‐transformed, the expression level of *CsNAC1* was significantly increased than in sample containing only the miR164d and *CsNAC1* expression vectors (Figure 3e). Furthermore, three vectors (35S::*CsNAC1*‐GUS, 35S::pre‐MIR164d, and 35S::lncRNA81246) were created for the GUS assay (Figure 3f), and the vectors were individually or co‐transformed into the leaves of *N. benthamiana*. A significant reduction in the staining area was found in leaves treated with the combination of *CsNAC1* and miR164d expression vectors. Conversely, there was a significant increase in the staining area in leaves treated with a mixture of *CsNAC1*, miR164d, and lncRNA81246 (Figure 3g). In conclusion, we determined that lncRNA81246 interacts with miR164d to interfere with the target interaction between miR164d and *CsNAC1*.

### MiR164d plays a positive role in tea plant disease resistance

**Figure 4:**
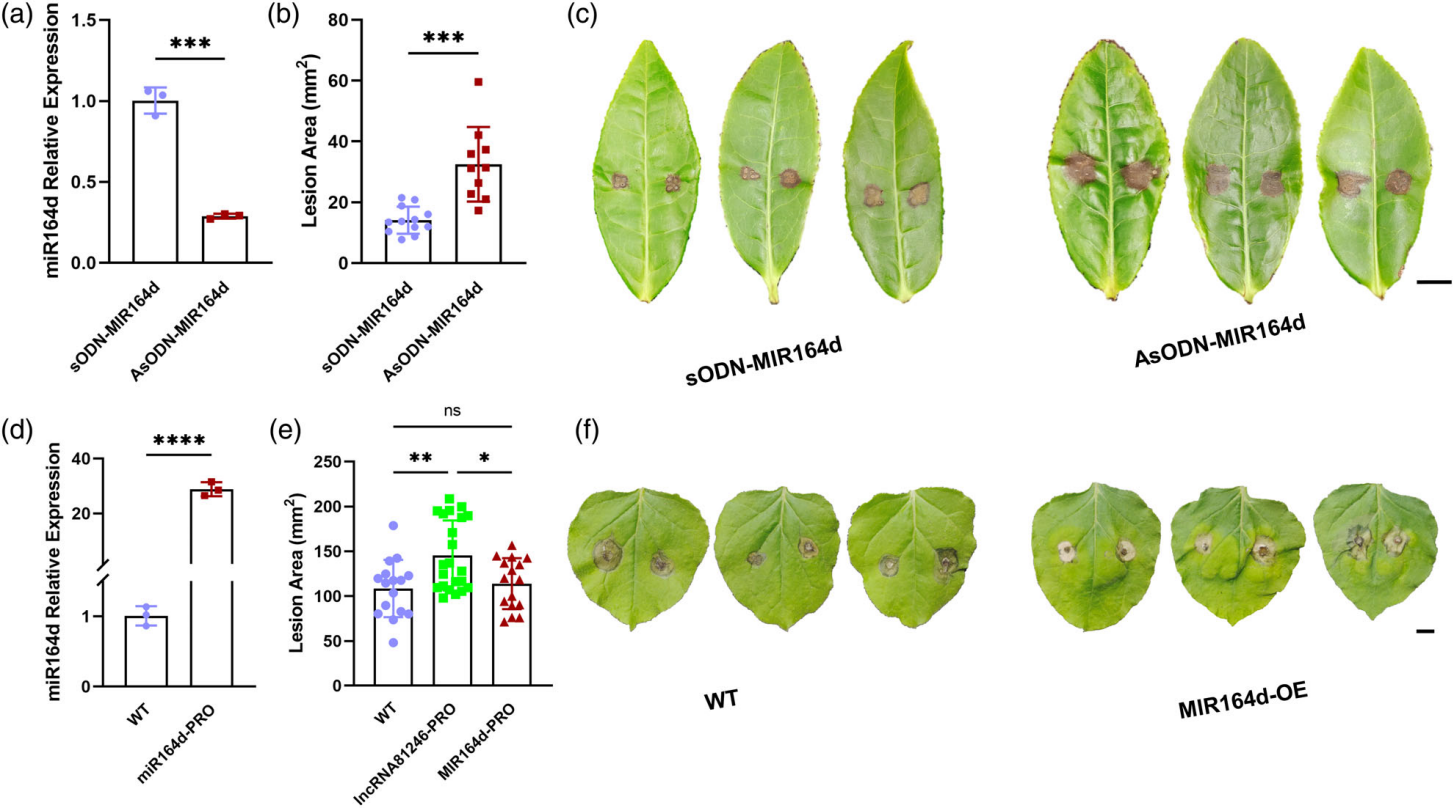
AsODN of MIR164d in tea leaves and transient MIR164d expression in tobacco leaves. (a) Relative expression levels of MIR164d after independent treatments with sODN‐MIR164d and AsODN‐MIR164d. (b) The lesion areas of tea leaves incubated independently with sODN‐MIR164d and AsODN‐MIR164d at 12 h after pathogen infection. (c) The phenotypes of tea leaves incubated independently with sODN‐MIR164d and AsODN‐MIR164d at 12 h after pathogen infection. Scale bar = 1 cm. (d) The relative expression levels of MIR164d in wild‐type and MIR164d‐overexpressed tobacco leaves. (e) The lesion areas in wild‐type and MIR164d‐overexpressed tobacco leaves at 12 h after pathogen infection. (f) The phenotypes of wild‐type and MIR164d‐overexpressed tobacco leaves at 12 h after pathogen infection. Scale bar = 1 cm. RT‐qPCR was normalized by the expression of *GAPDH* and U6 in tea plant and by U6 and *UBC* in tobacco. Error bars represent standard errors (*n* ≥ 3). Asterisks represent statistically significant differences (ns >0.05, *P < 0.05, **P < 0.01, ***P < 0.001, ****P < 0.0001) as determined by Student's *t*‐tests.

The negative regulation of disease resistance in tea plants by lncRNA81246 has been demonstrated. To investigate whether miR164d affects tea plant resistance, we silenced miR164d in tea leaves using AsODN and confirmed its successful silencing using RT‐qPCR (Figure 4a). At 12 h after inoculating *L. theobromae* hypha into the silenced tea leaves, we observed significant increases in the lesion areas compared with the control group treated with sODN (Figure 4b,c). To further verify the results of AsODN assay, we developed a 35S::pre‐miR164d‐overexpression vector and transformed it into tobacco using *Agrobacterium* GV3101. After 2 days, samples were collected, and the RT‐qPCR analysis showed a significant increase in miR164d expression (Figure 4d). Subsequently, we inoculated the transiently overexpressed plants with *B. cinerea* hypha and measured the lesion areas after 12 h. The heterologous transient overexpression of miR164d from tea greatly enhanced disease resistance in tobacco (Figure 4e,f). MiR164d may enhance the disease resistance of tea plants by degrading *CsNAC1* transcripts.

### *CsNAC1* negatively regulates tea plant disease resistance

**Figure 5:**
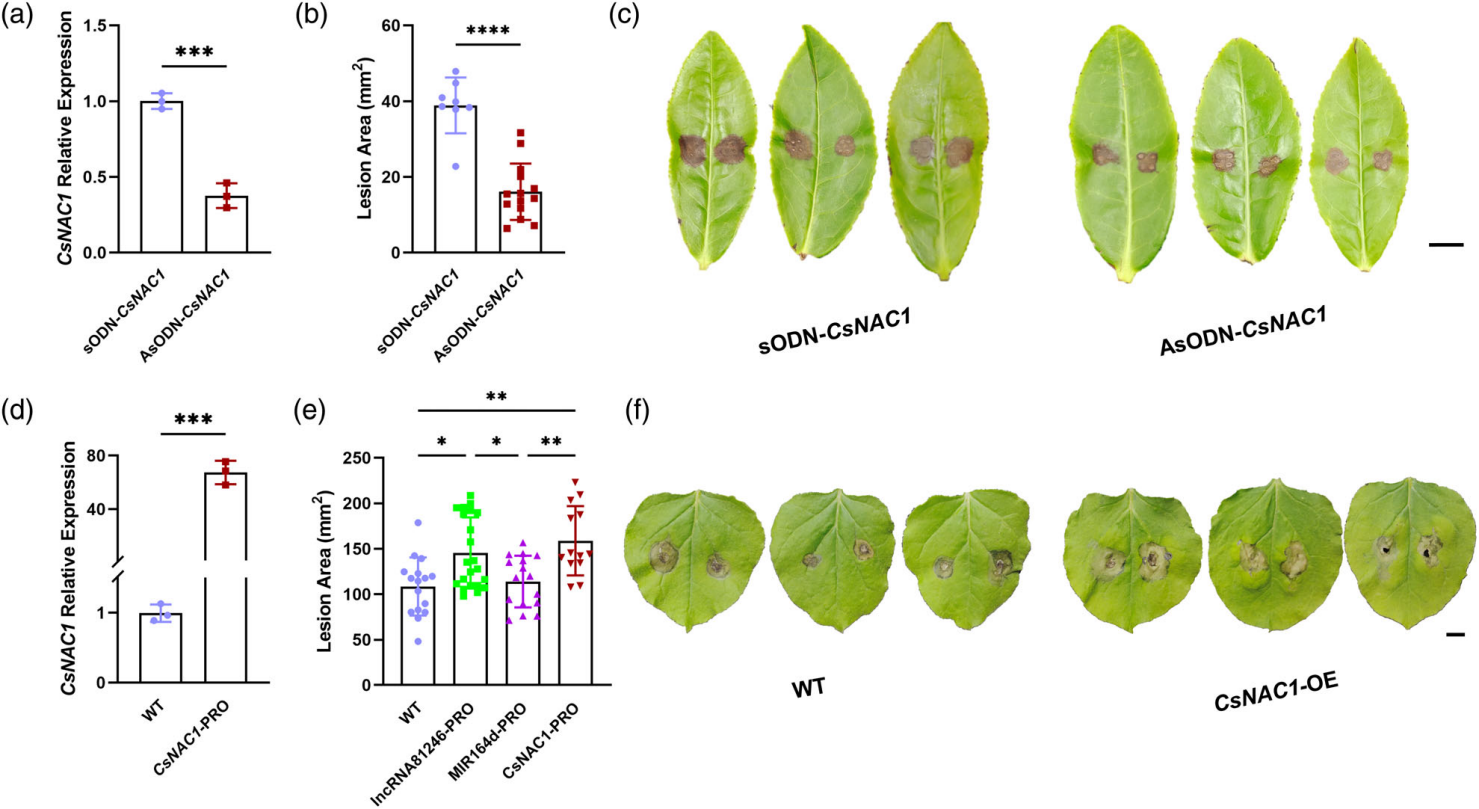
AsODN of *CsNAC1* in tea leaves and transient *CsNAC1* expression in tobacco leaves. (a) Relative expression levels of *CsNAC1* after independent treatment with sODN‐*CsNAC1* and AsODN‐*CsNAC1*. (b) The lesion areas of tea leaves incubated with sODN‐*CsNAC1* and AsODN‐*CsNAC1* at 12 h after pathogen infection. (c) The phenotypes of tea leaves incubated independently with sODN‐*CsNAC1* and AsODN‐*CsNAC1* at 12 h after pathogen infection. Scale bar = 1 cm. (d) The relative expression levels of *CsNAC1* in wild‐type and *CsNAC1*‐overexpressed tobacco leaves. (e) The lesion areas of wild‐type and *CsNAC1*‐overexpressed tobacco leaves at 12 h after pathogen infection. (f) The phenotypes of wild‐type and *CsNAC1*‐overexpressed tobacco leaves at 12 h after pathogen infection. Scale bar = 1 cm. RT‐qPCR was normalized by the expression of *GAPDH* in tea plant and by *UBC* in tobacco. Error bars represent standard errors (*n* ≥ 3). Asterisks represent statistically significant differences (*P < 0.05, **P < 0.01, ***P < 0.001, ****P < 0.0001) as determined by Student's *t*‐tests.

There is a significant differential expression of *CsNAC1* in tea plants infected with *L. theobromae* (Guo et al., 2022). To explore the function of *CsNAC1* in disease resistance, it was silenced in plants using AsODN. A RT‐qPCR analysis demonstrated a significant decrease in the expression of *CsNAC1* compared with plants receiving the sODN treatment (Figure 5a). Subsequently, tea leaves were inoculated with the *L. theobromae* hypha and lesion areas were measured after 12 h. The lesion areas on tea leaves showed significant reductions due to the decreased expression of *CsNAC1* (Figure 5b,c). Thus, CsNAC1 appears to be negatively involved in the regulation of disease‐resistance mechanisms in tea leaves. The role of the CsNAC1 in plant disease resistance was further investigated by introducing a 35S::*CsNAC1* vector into *N. benthamiana* via *Agrobacterium* GV3101, with the wild type serving as the control. After 2 days, the RT‐qPCR results for the treated samples demonstrated a more than 60‐fold increase in expression of *CsNAC1* compared with the wild type (Figure 5d). The leaves were then inoculated with the *B. cinerea* hypha. After 12 h, the lesion areas were measured, and the lesion areas of leaves overexpressing *CsNAC1* were significantly greater than those of WT (Figure 5e,f). Thus, these findings suggest that CsNAC1 exerts a negative regulatory effect on plant disease‐related defenses.

### CsNAC1 regulates *CsEXLB1* to promote pathogen infection

**Figure 6:**
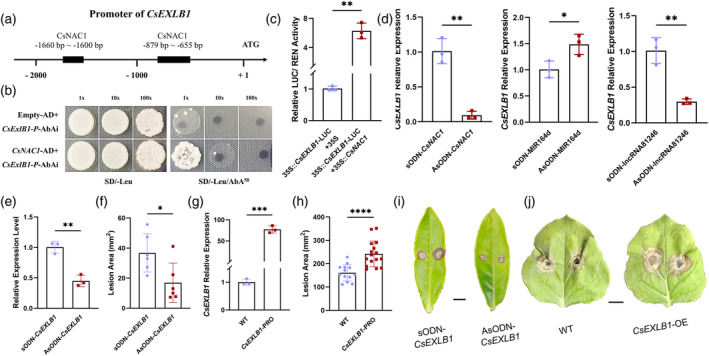
*CsNAC1* acts as a cis‐regulator of *CsEXLB1*. (a) Analysis of the *CsEXLB1* promoter, which contains two *CsNAC1*‐binding motifs. (b) Y1H assays indicated that *CsNAC1* binds to the *CsEXLB1* promoter. Yeast cells co‐transformed with pGADT7 + pAbAi::*ProCsEXLB1* were used as controls. The superscript numbers indicate the optimal AbA concentrations used for suppressing the background inositol phosphorylceramide biosynthesis of the pAbAi::*ProCsEXLB1* vector. (c) Effects of *CsNAC1* activation of the *CsEXLB1* promoter were determined using LUC/REN activity in *N. benthamiana* leaves. The LUC/REN ratio of the empty vector (SK) was used for normalization. (d) RT‐qPCR analysis of *CsEXLB1* in *CsNAC1*‐silenced, miR164d‐silenced, lncRNA81246‐silenced, and control tea leaves. (e) Relative expression levels of *CsEXLB1* after independent treatment with sODN‐*CsEXLB1* and AsODN‐*CsEXLB1*. (f) The lesion areas of tea leaves incubated independently with sODN‐*CsEXLB1* and AsODN‐*CsEXLB1* 12 h after pathogen infection. (g) The relative expression levels of *CsEXLB1* of wild‐type and *CsEXLB1*‐overexpressed tobacco leaves. (h) The lesion areas of wild‐type and *CsEXLB1*‐overexpressed tobacco leaves at 12 h after pathogen infection. (i) The phenotypes of tea leaves incubated independently with sODN‐*CsEXLB1* and AsODN‐*CsEXLB1 CsEXLB1* at 12 h after pathogen infection. Scale bar = 1 cm. (j) The phenotypes of wild‐type and *CsEXLB1‐*overexpressed tobacco leaves at 12 h after pathogen infection. Scale bar = 1 cm. RT‐qPCR was normalized by the expression of *GAPDH* in tea plants. Error bars represent standard errors (*n* ≥ 3). Asterisks represent statistically significant differences (*P < 0.05, **P < 0.01, ***P < 0.001, ****P < 0.0001) as determined by Student's *t*‐tests.

NAC is one of the largest transcription factor families in plants, we further demonstrated that CsNAC1 is a transcription factor in tea plants using the phylogenetic relationships and multiple sequence alignment of CsNAC1, and transcriptional activation assay (Figures S3, S4, S5) (Jeyaraj et al., 2019; Zhang et al., 2022). To investigate the regulatory mechanism of *CsNAC1*, we screened and analyzed the interactome map of CsNAC1 constructed in a previous study (Singh et al., 2021). Among the genes identified, *expansin‐like B1* (TEA012391) proved of particular interest, we then conducted a RT‐qPCR of *CsEXLB1* in tea leaves treated by AsODN‐CsNAC1. Upon treatment with AsODN‐*CsNAC1*, there was a decrease in the expression of *CsEXLB1*, which correlated with the decrease in *CsNAC1* expression (Figure 6d). Additionally, we performed RT‐qPCR assays on tea leaves treated with AsODN‐lncRNA81246 and AsODN‐miR164d to examine their effects on *CsEXLB1*. Silencing miR164d led to an increase in the expression level of *CsEXLB1*, consistent with the effect observed for *CsNAC1*. Conversely, silencing lncRNA81246 resulted in decreased *CsEXLB1* (Figure 6d). NAC transcription factor can bind cis‐acting elements with similar core sequences “CACG” or “CGT[G/A]” (He et al., 2018). Further examination of the promoters of *CsEXLB1* revealed “CACG” or “CGT[G/A]” distributed in two fragments located 655–879‐bp and 1600–1660‐bp upstream of the transcription start site (Figure 6a). Subsequently, we cloned the two fragments containing NAC‐binding motifs and ligated them into the pAbAi vector, as well as cloning *CsNAC1* into pGADT7. Yeast one‐hybrid (Y1H) assays were utilized to confirm the regulatory relationship between CsNAC1 and fragments of *CsEXLB1*. CsNAC1 was capable of binding to the promoter fragments located 655–879 bp of the transcription start site of *CsEXLB1* (Figure 6b). Then, dual‐luciferase assays were employed to confirm the regulatory relationship between CsNAC1 and *CsEXLB1*. After co‐expression of CsNAC1, we found a significant increase in luciferase activity (Figure 6c). The results suggest that CsNAC1 regulates the expression of *CsEXLB1*.

**Figure 7:**
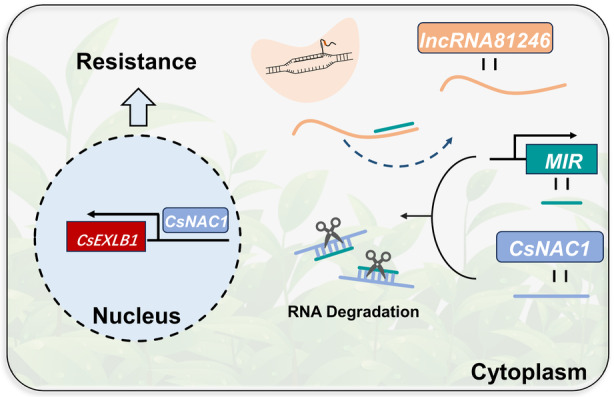
Proposed model in which lncRNA81246 interferes with the miR164d‐guided cleavage of mRNAs of *CsNAC1* to regulate resistance to tea leaf spot.

AsODN and transient overexpression assays were conducted to determine the function of *CsEXLB1*. After silencing *CsEXLB1* with AsODN, its expression was significantly reduced as determined by RT‐qPCR analysis when compared with expression after the sODN treatment (Figure 6e). In addition, the lesion area was significantly decreased after treatment with AsODN‐*CsEXLB1* (Figure 6f,i). Transient expression of *CsEXLB1* in tobacco significantly increased the *CsEXLB1* expression level compared with the WT (Figure 6g). Additionally, the lesion areas of the overexpressing tobacco were observed to be significantly greater than those of the wild type (Figure 6h,j). These results indicated CsEXLB1 is a negative regulator against tea leaf spot. In conclusion, CsNAC1 is involved in tea plant resistance to tea leaf spot disease by regulating the expression of *CsEXLB1* (Figure 7).

## DISCUSSION

Our previous study identified a ceRNA regulatory module dominated by lncRNAs in response to tea leaf spot infection (Guo et al., 2022). We observed a significant reduction in the expression level of lncRNA81246, suggesting its potential involvement in the immune regulation of tea plants (Table S1). In this study, we identified lncRNA81246 acts as an ncRNA through bioinformatics analyses and GUS histochemical staining assays (Figure 1a–c). Cytoplasmic lncRNAs possess the capability to directly bind with miRNAs and function as miRNA sponges, participating in the regulation of biological processes (Thomson & Dinger, 2016). We firstly found that lncRNA81246 exists in both the nucleus and cytoplasm based on nuclear and cytoplasmic extract assays, indicating its ceRNA regulatory potential (Figure 1d–e). Furthermore, after silencing lncRNA81246, we confirmed its negative regulation on disease resistance in tea plant (Figure 2a–c). Additionally, because of the high homology between miR164d in various plant included tea plant, we overexpressed lncRNA81246 in tobacco and observed a significant decrease in immunity (Figure 2d–f; Figures S1, S2). Although lncRNAs are not conserved, many previous studies have utilized heterologous expression as a method to investigate the functions of lncRNAs due to the high conservation of associated miRNAs (Du et al., 2018; Yang et al., 2019). We subsequently validated a novel direct interaction between lncRNA81246 and miR164d using GUS staining and a dual‐luciferase assay in tobacco leaves transiently co‐expressing lncRNA81246, miR164d, and *CsNAC1* (Figure 3a,d–g). Silencing lncRNA81246 in tea leaves resulted in a significant increase in miR164d expression and a decrease in *CsNAC1* and *CsEXLB1* expression levels, indicating its novel and significant regulatory role in tea plant resistance against *L. theobromae* (Figure 3a).

Numerous studies on *Arabidopsis*, rice, and wheat (*Triticum aestivum* L.) have demonstrated that miRNA164 plays a prominent role in regulating plant immunity against pathogens (Wang et al., 2018; Zhou et al., 2023; Lin *et al*., 2022; Zhan et al., 2021). Similarly, miRNA164 family members mediate immunity against anthracnose in tea plants by targeting the NAC transcription factors and inhibiting their expression (Jeyaraj et al., 2019). In this study, we performed degradome sequencing and modified 5′ RLM‐RACE assays to validate miR164d and *CsNAC1* cleavage sites. The cleavage site is in the seed region (9th–11th nucleotides) of miR164d (Figure 3b,c). In addition, GUS histochemical staining assays confirmed that miR164d suppressed *CsNAC1* expression (Figure 3g). Transient co‐expression experiments in tobacco showed that overexpression of miR164d significantly decreased *CsNAC1* expression (Figure 3e). Furthermore, samples in which miR164d had been silenced using AsODN showed significant increases in *CsNAC1* expression (Figure 3a). Thus, these experiments demonstrated that *CsNAC1* is a target of miR164d. Tea leaves in which miR164d was silenced showed increased lesion areas (Figure 4a–c), whereas the transient overexpression of miR164d significantly improved tobacco disease resistance (Figure 4d–f). Therefore, miR164d is a positive regulator in tea plant resistance.

NAC transcription factors regulate plant immunity to pathogen by bridging signaling pathways between plant hormones (Bian et al., 2020; Lee et al., 2017). In this study, the inhibition of *CsNAC1* expression via AsODN in tea plants enhanced their resistance to pathogen infection (Figure 5a–c). Transient overexpression of *CsNAC1* in tobacco increased its susceptibility to the pathogen (Figure 5d–f). The results indicated that CsNAC1 negatively regulates the disease resistance of tea plants. Consistent with this study, a knockdown of *GhNAC100* enhances plant resistance to cotton yellow wilt fungus (Hu et al., 2020). Silencing *TaNAC1*, *TaNAC21*/22, or *TaNAC30* enhances wheat resistance to *Puccinia striiformis f*. sp. *Tritici* (Feng et al., 2014; Wang et al., 2015; Wang, Wei, et al., 2018). Here, we identified CsNAC1 as a regulator of *CsEXLB1* expression using Y1H and dual‐luciferase assays (Figure 6d–f). A RT‐qPCR analysis of AsODN‐*CsNAC1*‐silenced tea leaves also demonstrated a significant decrease in the *CsEXLB1* expression level (Figure 6a–c). CsEXLB1 is an expansin‐like protein. The overexpression of expansin proteins in rice increases cell wall loosening, facilitating pathogen infection (Ding et al., 2008). In *Arabidopsis*, *AtEXLA2* mutant plants have enhanced resistance against the pathogens *B. cinerea* and *Alternaria brassicicola* (Abuqamar et al., 2013). Although *OsEXPA10* promotes rice growth, it also increases susceptibility to brown planthopper and blast attack (Tan et al., 2018). Thus, we concluded that CsNAC1 weakened tea plant resistance by upregulating *CsEXLB1* expression.

In sum, we propose a ceRNA regulatory module to explain how the lncRNA81246–miR164d interaction affects tea plant resistance. The lncRNA81246 binds efficiently to miR164d and attenuates the miR164d‐mediated silencing of *CsNAC1*. In addition, this binding decreased miR164d expression, resulting in an increase in the expression level of the target gene *CsNAC1*, thereby partially regulating the expression of *CsEXLB1* and negatively regulating the resistance of tea plants. Our research represented a new understanding of post‐transcriptional regulatory mechanisms governed by lncRNAs and revealed an important role played by the lncRNA81246–miR164d–*CsNAC1* network in modulating plant defense responses.

## MATERIALS AND METHODS

### Plant material and treatments

Three‐year‐old tea plants were grown in greenhouse under the conditions described above (Li et al., 2019). Tobacco was cultivated in a greenhouse with long‐day (16 h of light and 8 h of dark) conditions at 24°C with 70%–80% relative humidity. The pathogen of *L. theobromae* strain CGMCC 3.20151 and *B. cinerea* strain CGMCC3.20932 were grown on potato dextrose agar, and pathogenicity was determined in accordance with the methods of our research laboratory. Each experiment was independently replicated three times.

### Total RNA extraction and RT‐qPCR

Total RNA was extracted using the TransZol Up Plus RNA Kit (TransGen, Beijing). First‐strand cDNA synthesis was performed using an EasyScript One‐Step gDNA Removal and cDNA Synthesis SuperMix Kit (TransGen, Beijing). RT‐qPCR was performed using Premix Ex Taq™ (TaKaRa, Japan). Gene expression levels were analyzed using gene‐specific primers. Relative gene expression was calculated using the 2^−ΔΔCt^ method (Livak and Schmittgen, 2001).

### Bioinformatics analyses of *CsNAC1*

Briefly, ClustalW software (https://www.genome.jp/tools‐bin/clustalw) was used for multiple sequence alignments. A phylogenetic tree was constructed using the MEGAX (https://www.megasoftware.net/) proximity method (Neighbor joining) with 1000 bootstrap replications. Protein sequences were compared, and domains were analyzed, using GeneDoc software (http://nrbsc.org/gfx/genedoc/index.html).

### Degradome sequencing

Degradome sequencing was performed as described previously with minor modifications (Lin et al., 2016). Total RNA from control and infected leaves were used to construct two degradome libraries (CK treatment and Infected treatment). ACGT101‐DEG (LC Sciences, https://www.lcsciences.com/) and CleaveLand 3.0 software packages were used to detect possible miRNA targets (https://sites.psu.edu/axtell/software/cleaveland3/). All the targets were divided based on their abundance.

### 5′ RLM‐race

The 5′ RLM‐RACE was performed as described previously (Wang et al., 2021). The 5′ RNA adaptor was ligated using T4 RNA ligase. After synthesizing the cDNA, the gene‐specific primers and adaptor primer were used to amplify the products (Table S2).

### Antisense oligonucleotide

The Soligo online tool (https://sfold.wadsworth.org/cgi‐bin/soligo.pl) was used to select antisense oligonucleotides (Ding and Lawrence, 2003). AsODN was performed as described previously (Hu et al., 2022; Li, Li, et al., 2022; Li, Ye, et al., 2022). In brief, tea shoots having one bud and two leaves were cut and placed in Eppendorf tubes containing 1 mL of 20‐μM AsODN‐*CsNAC1*, AsODN‐MIR164d, and AsODN‐lncRNA81246. The sense oligonucleotides acted as controls.

### Transient expression in *N. benthamiana*

The CDSs of *CsNAC1*, miR164d precursor sequence, lncRNA81246, and *CsEXLB1* were individually inserted into pBI121 and transferred into *Agrobacterium* GV3101 (listed in Table S2). Leaves were harvested 2 days after infiltration for experiments.

### Transactivation activity assay

The CDS of *CsNAC1* was inserted into pGBKT7. Subsequently, yeast strain AH109 was used to generate pGBKT7‐*CsNAC1* and pGBKT7 vectors. Finally, following positive PCR detection, individual colonies of positive clones were selected and grown in the selective media SD/−Trp, SD/−Trp − Ade, and SD/−Trp − Ade/X‐a‐gal in the dark at a constant 30°C for 3 days. Primers used are listed in Table S2.

### Yeast one‐hybrid assay

The CDS of *CsNAC1* was inserted into pGADT7. Two fragments of the *CsEXLB1* promoter contain cis‐regulation elements of CsNAC1 was cloned into the pAbAi vector. The construct was then transformed into the yeast strain Y1H Gold and spread over SD/−Ura selective medium (Clontech, Mountain View, CA, USA). pGADT7‐*CsNAC1* was transformed into Y1H Gold harboring the pAbAi vector fused with the promoter region of *CsEXLB1* and spread on SD/−Leu selective medium containing the selected AbA concentration. P53‐AbAi was used as a positive control. The protocol was performed as described previously (Liu et al., 2021). Primers used are listed in Table S2.

### Dual luciferase reporter assay

The CDSs of *CsNAC1* and two fragments of the *CsEXLB1* promoter containing cis‐regulation elements of CsNAC1 were inserted into pGreenII 62‐SK and pGreenII 0800‐LUC. To verify the interaction between lncRNA81246 and miR164d, their sequences were cloned into pGreenII 0800‐LUC and pGreenII 62‐SK using specific primers (listed in Table S2). The recombinant plasmids were then transformed into *A. tumefaciens* strain GV3101. The cultures were mixed (10:1 v/v) and then infiltrated into 28‐day‐old leaves of *N. benthamiana*. Firefly luciferase and Renilla (REN) luciferase enzyme activities were tested using a Dual‐Luciferase Reporter Assay Kit (Yeasen, China), and LUC/REN ratio was used as evaluate the expression level.

### Nuclear and cytoplasmic extract assay

Nuclear and cytoplasmic extraction assays were performed as previously described (Zhang et al., 2021). Briefly, the tea leaves were ground into a fine powder and mixed with 2 mL/g lysis buffer. After centrifugation at 10000*g* for 10 min at 4°C, the supernatant was collected as a cytosolic extract. The pellet was washed four times with NRBT buffer. Then, the pellet was resuspended in NBR2 buffer. The mixed solution was carefully added to the surface layer of NRB3 Buffer and centrifuged at 16000*g* for 45 min at 4°C. The final precipitate was resuspended with lysis buffer to obtain the nuclear extract. U6 RNA was used as a nuclear marker, and *β*‐*actin* was used as a cytoplasmic marker.

### Histochemical GUS staining

The CDSs of *CsNAC1*, miR164d precursor, and lncRNA81246 were independently inserted into the pBI121 vector. Tissues were stained with GUS as previously described (Jefferson et al., 1987). A minimum of three experiments was performed, and the pBI121‐GUS vector was used as a control. Primers are listed in Table S2.

## Author Contributions

DG conceived the experiment and analyzed the data. DG and DXL preformed the assays. DG, FL, J‐JZ, and YM wrote the manuscript. BS and ZC designed the experiments, conducted the whole study, and edited the article. All authors have read and agreed to the published version of the manuscript.

## Conflicts of Interest

The authors declare no conflicts of interest.

## Supporting information


**Figure S1.** The conservation of the miR164 family from tea plant.

**Figure S2.** Multiple sequence alignment of miR164d from different species.

**Figure S3.** The phylogenetic relationships of *CsNAC1*.

**Figure S4.** Multiple sequence alignment of *CsNAC1* homologous genes between tea plant and other species.

**Figure S5.** Transcriptional activation analysis of *CsNAC1*.

**Figure S6.** Predicted base‐pairing between lncRNA81246 and miR164d.

**Table S1.** Analysis the results of whole transcriptome sequencing and degradome sequencing among lncRNA81246, miR164d, and *CsNAC1*. And the predicted results of the ceRNA regulate module mediated by lncRNA81246, miR164d, and *CsNAC1*.
**Table S2.** All primers or oligonucleotides used in this study.
**Table S3.** Sequences of all genes in this study.
**Table S4.** The predict results of interactome map of CsNAC1.

## Data Availability

The article's supporting data and materials can be found within the manuscript and its supporting materials.
