# Supplementary figures and images for: LncRNA81246 regulates resistance against tea leaf spot by interrupting the miR164d‐mediated degradation of NAC1
Source: Plant J. 2024 Nov 26;121(1):e17173. doi: 10.1111/tpj.17173 (PMC11711933; doi:10.1111/tpj.17173)

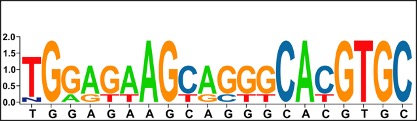

Supplement: Supplementary file 1 — Figure S1. The conservation of the miR164 family from tea plant. [file TPJ-121-0-s006.jpg]

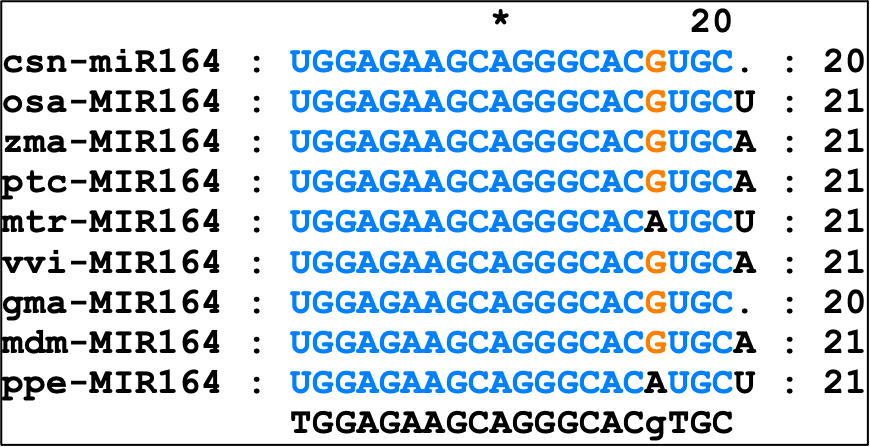

Supplement: Supplementary file 2 — Figure S2. Multiple sequence alignment of miR164d from different species. [file TPJ-121-0-s007.png]

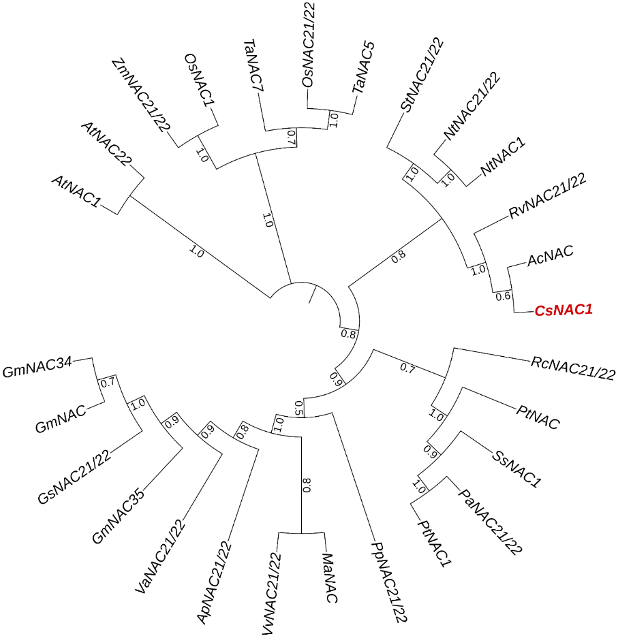

Supplement: Supplementary file 3 — Figure S3. The phylogenetic relationships of CsNAC1. [file TPJ-121-0-s003.png]

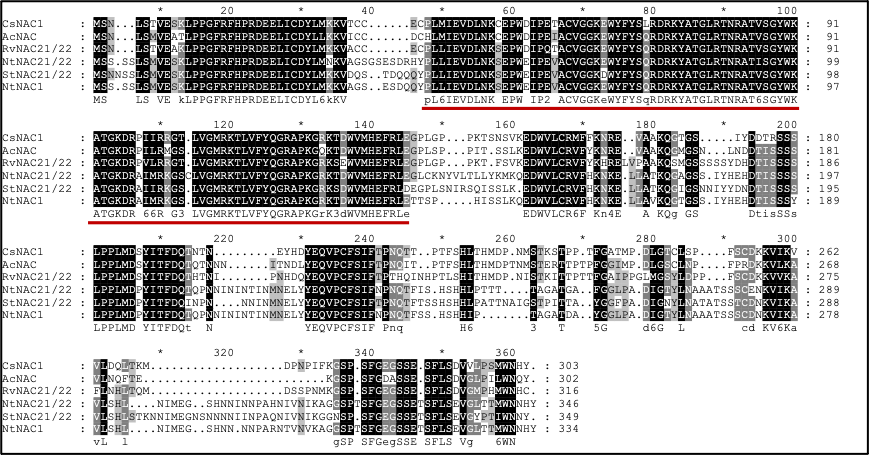

Supplement: Supplementary file 4 — Figure S4. Multiple sequence alignment of CsNAC1 homologous genes between tea plant and other species. [file TPJ-121-0-s004.png]

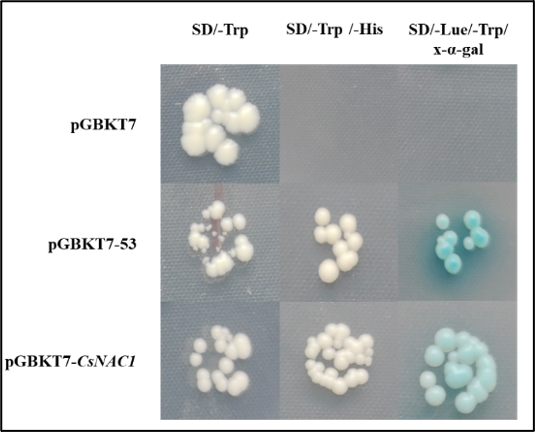

Supplement: Supplementary file 5 — Figure S5. Transcriptional activation analysis of CsNAC1. [file TPJ-121-0-s001.png]

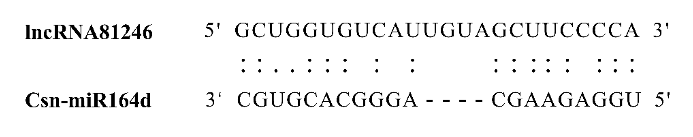

Supplement: Supplementary file 6 — Figure S6. Predicted base‐pairing between lncRNA81246 and miR164d. [file TPJ-121-0-s005.png]
